# Evaluation of a Three-Marker Panel for the Detection of Uveal Melanoma Metastases: A Single-Center Retrospective Analysis

**DOI:** 10.3390/cancers13102464

**Published:** 2021-05-18

**Authors:** Zenan Lin, Daniela Süsskind

**Affiliations:** University Eye Hospital, Center for Ophthalmology, University of Tübingen, 72076 Tübingen, Germany; daniela.suesskind@med.uni-tuebingen.de

**Keywords:** uveal melanoma, metastasis, multi-biomarker panel, B-cell activating factor, growth differentiation factor-15, osteopontin

## Abstract

**Simple Summary:**

Blood-based B-cell activating factor (BAFF), growth differentiation factor-15 (GDF-15) and osteopontin (OPN) have been reported to be biomarkers for the uveal melanoma (UM) metastases. This work intended to assess their kinetics and to evaluate their significance as a three-marker panel for clinical practice. Our results not only provided their cutoff values for differentiating the metastatic patients from non-metastatic patients, but also confirmed that the three-marker panel outperformed any single biomarker in distinguishing metastatic patients. Besides, the increasing trends of the levels of three biomarkers were detected in the two-year period before the imaging diagnosis of metastases. The multiplex panel of BAFF, GDF-15 and OPN might be a utilizable implementation for the detection of UM metastases. Since it is a retrospective pilot work, more well-designed prospective studies employing larger cohorts are still needed to validate the findings.

**Abstract:**

Blood-based B-cell activating factor (BAFF), growth differentiation factor-15 (GDF-15) and osteopontin (OPN) have been identified to be promising biomarkers for the metastases of uveal melanoma (UM). This study intended to assess their kinetics and to evaluate their significance as a three-marker panel. A group of 36 UM patients with and 137 patients without metastases were included in the study. Their plasma OPN levels were measured by ELISA; serum BAFF and GDF-15 levels were determined with a Luminex MAGPIX system. Receiver operating characteristic (ROC) analysis was performed to calculate the cutoff values of the three markers for identifying the patients with metastases. The ability to identify patients with metastases was compared between the single markers and the combination as a three-marker panel. By using the Student’s *t*-test, we also investigated the kinetic changes of the levels of BAFF, GDF-15 and OPN across six periods (i.e., 0–6 months, 6–12 months, 12–18 months, 18–24 months, >24 months and post-metastasis) before the imaging diagnosis of metastases. By maximizing the Youden’s index, the serum GDF-15 level of 1209 pg/mL and the plasma OPN level of 92 ng/mL were identified to have the best performance for distinguishing the metastatic patients from non-metastatic patients. The three-marker panel offered a better performance in distinguishing patients with metastases, with an area under the curve of 0.802, than any single biomarker. Increasing trends of the levels of three biomarkers were observed in the two-year period before the imaging diagnosis of metastases. The combined panel of BAFF, GDF-15 and OPN might be a utilizable implementation for the detection of UM metastases. In the bioinformatics study with two external datasets, the high expression of gene *BAFF* and *GDF-15* in primary UM tissues was identified to be associated with poor overall survival rates. As the current work is a single-center retrospective study, more well-designed prospective investigations employing larger cohorts are urgently needed to validate our findings.

## 1. Introduction

As the most common primary intraocular malignancy, uveal melanoma (UM) was reported to have an incidence of six per one million in America and Europe [1]. Thanks to medical advancements, various forms of therapeutic eye preserving approaches such as radiotherapy and local resection are available to treat the primary UM. Unfortunately, half of UM patients were estimated to develop fatal metastatic diseases with an obvious organ preference of the liver. So far, despite the surgical interventions for resectable metastatic lesions, few conventional therapeutic methods were found to be effective in treating the metastatic UM [1]. To make matters worse, novel treatments such as chemotherapies and immune checkpoint inhibitors, which were effective for metastatic cutaneous melanoma, demonstrated poor outcomes for metastatic UM [1,2]. Therefore, an early diagnosis of the metastases is assumed to allow an on-time surgical resection and provide better chances of curing.

Currently, imaging modalities such as computer tomography (CT), magnetic resonance imaging (MRI), ultrasonography, and Liver Functional Tests (LFTs) are widely used to detect the metastatic UM in the clinic. However, each of them had its limitations to some extent, such as cumulative radiation exposure for CT, high expense for CT and MRI, limited resolution for ultrasonography and low sensitivity for LFTs [3,4]. To overcome the disadvantages of conventional screening methods of UM patients, researchers are embarking on finding new methods for monitoring the metastatic risk of UM indicating better sensitivity and specificity.

Therefore, investigators started to look for new and better screening and monitoring methods and focused on easily accessible blood samples with the aim to find potential blood-based biomarkers for the screening of UM metastases [5,6]. So far, the explorations of the blood of UM patients have brought fascinating discoveries [5,6]. In several independent investigations of UM, a variety of proteins were identified to be promising metastasis-associated biomarkers [5,6]. For instance, the upregulated blood-based expression of osteopontin (OPN), S-100β, growth differentiation factor-15 (GDF-15), melanoma inhibitory activity (MIA) and B cell-activating factor (BAFF) were confirmed to have associations with the metastases of UM [7,8,9,10,11,12].

Given the fact that cancer is a complex disease and no single unique biomarker is sufficient enough to reflect the metastatic status, the assessment of multiple markers might provide more complete and valuable information [9,13]. Previous observations on melanoma have confirmed that screening panels consisting of multiple biomarkers were superior to the application of a single biomarker since the multiplex panels would offer higher sensitivities and specificities to detect the metastases [14,15]. In particular, Barak and colleagues have already examined the serum levels of OPN, S-100β and MIA, and found that the combination of these three markers would outperform single markers in distinguishing the UM patients with metastases from those without [14]. In another study, they also observed the increasing trends of these markers before the confirmation of metastases by imaging modalities and biopsy [16].

Therefore, in the current work, our previous data of BAFF will be integrated and analyzed with GDF-15 and OPN to determine whether this multiplex assay would improve the predictive power in comparison to focusing on a single biomarker. Besides, we also aimed to evaluate the kinetic development of the blood-based levels of BAFF, OPN and GDF-15 and compare their performance in detecting the metastases versus the conventional imaging diagnostic modalities.

## 2. Methods

### 2.1. Patient Enrollment and Eligibility Criteria

A total of 173 UM patients (36 patients with and 137 without metastases) treated in our eye hospital were included in this work. In brief, we only included the adult UM patients who were able to give consent to join in this project. Additionally, the patients with an active second malignancy were regarded as ineligible and dismissed from further analysis. The detailed clinicopathological features of the included patients were summarized in our previous report [9]. No remarkable differences in the age, sex, treatment methods and categories of tumor-infiltrating lymphocytes were observed between metastatic and non-metastatic patients [9]. Besides, 23 healthy individuals and their blood specimens were acquired after the consent was approved. The controls had an age range from 22 to 78 years (median: 49 years old) [9]. Our study was conducted under the guidance of the Declaration of Helsinki as revised in Tokyo and Venice and was approved by the local ethics committee (449/2018BO2) [9].

### 2.2. Blood Acquisition

Four blood samples (each 7.5 mL) were taken at each patient visit in the working hours during daytime. Two blood samples were allowed to stand for 30 min. Following this, they were centrifuged for 30 min at 2200× *g*. The supernatant was distributed into 1.5-mL microfuge tubes and centrifuged for a second time to remove most erythrocytes. The supernatant (serum) was frozen in 0.5-mL aliquots at −80 °C. The other two blood samples were distributed into Li-Heparin-containing tubes. They were centrifuged for 30 min at 2200× *g*. In order to deplete the platelets, the supernatant was aspirated into 1.5-mL microfuge tubes for the second centrifugation. The supernatant (plasma) was also apportioned into 0.5-mL aliquots and stored at −80 °C in the refrigerator.

### 2.3. Determination of BAFF Serum Concentration

The determination of the BAFF serum concentration has been described in our previous publication [9].

### 2.4. Determination of GDF15 Serum Concentration with Luminex Assay

Serum samples were diluted and examined in duplicate per each assay according to the manufacturer’s instructions. Then, GDF-15 levels were analyzed by the Human Premixed Multi-Analyte Kit (Kit Lot Number: L123335, R&D Systems, Minneapolis, MN, USA) of Magnetic Luminex Assay (Kit Catalog Number: LXSAHM-03, R&D Systems, Minneapolis, MN, USA) following the manufacturer’s instructions. Quantitative evaluation of serum GDF-15 levels was conducted in a multiplexed sandwich enzyme-linked immunosorbent assay (ELISA) system (Luminex MAGPIX system, Luminex Corporation, Austin, TX, USA). A 5-parameter logistic curve-fitting method was introduced to calculate the concentrations of GDF-15. We also recorded the time points at which sera were acquired.

### 2.5. Determination of OPN Plasma Concentration with ELISA

Previously, a study on epithelial malignant pleural mesothelioma indicated that the levels of plasma OPN are more stable than serum OPN [17]. Therefore, in this work, we measured the concentration of plasma OPN instead of serum OPN. The commercially available ELISA kit (Cat No.: DOST00, R&D Systems, Minneapolis, MN, USA) was used to test the plasma OPN levels in this study. The test was executed according to the manufacturer´s instructions. Finally, a reader (Infinite 200, TECAN, Zürich, Switzerland) which had the wavelength set to 450 nm and the corrected wavelength set to 540 nm was used to measure the optical densities. Then, we generated the standard curve by using a 4-parameter logistic curve-fitting method and calculated the plasma OPN levels of tested samples according to the manufacturer’s guidance.

### 2.6. Bioinformatics Study with Two Publicly Available Datasets on UM

Here, two publicly available gene expression profiles of UM were downloaded for survival analysis. With R (version 3.6.3) package TCGAbiolinks (version 2.12.6), we downloaded the gene expression and survival data of 80 primary UM samples from the TCGA uveal melanoma cohort (TCGA-UVM) [18,19]. The gene sequencing data were transformed to log_2_^(Transcripts Per Million+1)^ for further analysis. We also used the R package GEOquery (version 2.54.1) to acquire the gene sequencing (robust spline normalization values) and survival data of 28 primary UM tissues from the Gene Expression Omnibus (No.: GSE84976) [20,21].

In each cohort, the patients were ordered according to the gene expression of *BAFF*. Those with *BAFF* expression higher than the median value were labeled with “High expression of BAFF”, while the others were labeled with “Low expression of BAFF”. The same approach was applied to divide the patients according to the gene expression of *GDF-15* and *OPN*.

### 2.7. Statistics

Statistical analysis was conducted with the help of JMP^®^ (version 15.1.0, SAS Institute Inc., Cary, NC, USA), GraphPad Prism (version 6.0c, GraphPad Software, San Diego, CA, USA) and SPSS (version 25, IBM, Armonk, NY, USA). For all statistical evaluations, a *p* less than 0.05 was regarded as statistically significant. The levels of three makers were tested in the same cohort. As the original data was abnormally distributed, we conducted the natural logarithm transformation of the blood-based levels of these cytokines and then performed the one-way analysis of variance (ANOVA) and Tamhane post hoc analysis to compare them between 23 healthy controls, 36 patients with metastases and 137 patients without metastases. The first serum specimen in the non-metastatic patients and the first serum sample after diagnosis of metastases in the metastatic patients were evaluated in this regard. The first serum samples from the metastatic patients were all acquired within the first year after the diagnosis of metastases, with a mean time interval of 2.5 months. Pearson’s correlation coefficient was conducted to evaluate the associations between the metastatic burden and the levels of BAFF, GDF-15 and OPN. Receiver operator characteristic (ROC) analysis was introduced to calculate the area under the curve (AUC) for a single marker as well as for the combination of three markers. Additionally, Pearson’s correlation coefficient was also performed to assess the associations between the levels of BAFF, GDF-15 and OPN. According to the methods described by Barak et al. [16], we applied the Student’s *t*-test to compare the blood-based levels of three markers at the time periods of 0–6, 6–12, 12–18, 18–24 and >24 months before the confirmation of metastases by imaging modalities. Besides, Kaplan–Meier analyses were conducted to explore cohorts TCGA-UVM and GSE84976, and determine the prognostic differences between the groups of high- and low-expression of three markers. The log-rank test was used to examine the *p-value*.

## 3. Results

### 3.1. The Blood-Based Levels of GDF-15 and OPN in Different Patient Cohorts

**Figure 1:**
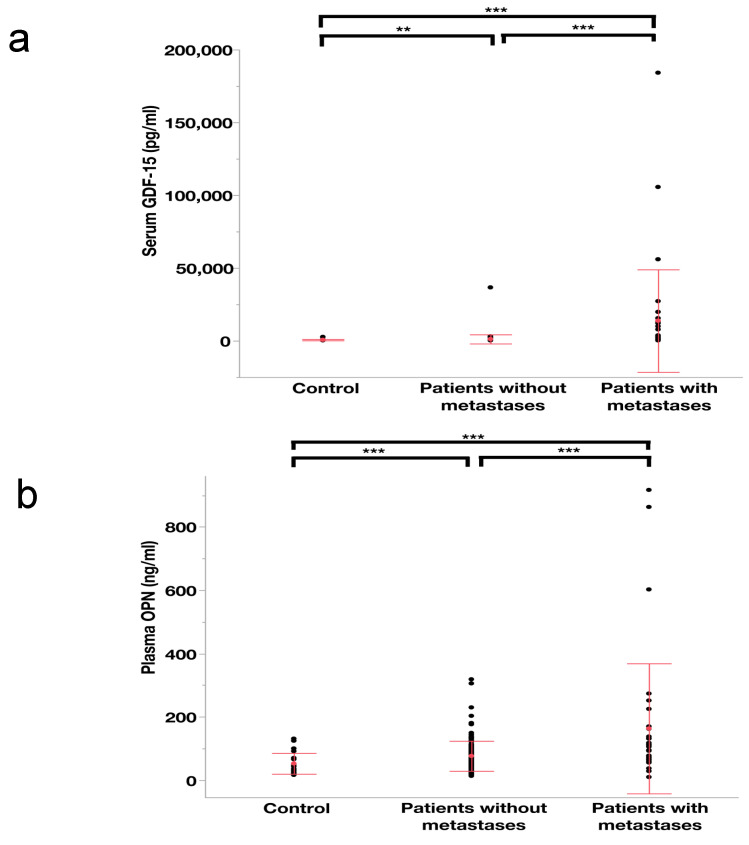
Scatter plots showing the distribution of blood-based levels of GDF-15 (**a**) and OPN (**b**) with mean (red point) and standard deviation (red lines) in control individuals, patients without and those with metastases. A one-way ANOVA and Tamhane post hoc analysis were conducted on the ln^(Serum GDF−15)^ and ln^(Plasma OPN)^, respectively. Note: *** means *p* < 0.01, ** means *p* < 0.05.

The first serum specimen in the non-metastatic patients and the first serum sample after diagnosis of metastases in the metastatic patients were analyzed here. According to our previous work on BAFF [9], the serum BAFF concentrations (Mean ± SD) in control individuals, patients with and without metastases were 810.3 ± 140.5 pg/mL, 1520.8 ± 1182.1 pg/mL and 950.4 ± 494.6 pg/mL, respectively. We found that the ln^(serum BAFF)^ levels were significantly higher in metastatic patients than in those without metastases and healthy controls [9]. The serum GDF-15 levels (Mean ± SD) in healthy controls, metastatic patients and non-metastatic patients were 643.6 ± 465.1 pg/mL, 13,750.7 ± 35,195.4 pg/mL and 1161.4 ± 3137.9 pg/mL, respectively. The results suggested that the patients with metastases had significantly higher levels of ln^(serum GDF−15)^ than those without metastases (see Figure 1a). Similarly, the levels of ln^(plasma OPN)^ were also remarkably higher in the metastatic patients than in those without metastatic lesions (see Figure 1b). The plasma OPN levels of healthy individuals, patients with and those without metastases were 52.7 ± 32.9 ng/mL, 163.3 ± 205.3 ng/mL and 76.6 ± 47.4 ng/mL, respectively. Notably, in comparison with healthy subjects, the UM patients without metastases were identified to have significantly higher concentrations of both GDF-15 and OPN (see Figure 1a,b).

### 3.2. Correlations between the Blood-Based Levels of BAFF, GDF-15 and OPN and the Metastatic Burden

In our previous publication, we have already observed a moderate correlation between BAFF serum levels and the metastatic burden [9].

**Figure 2:**
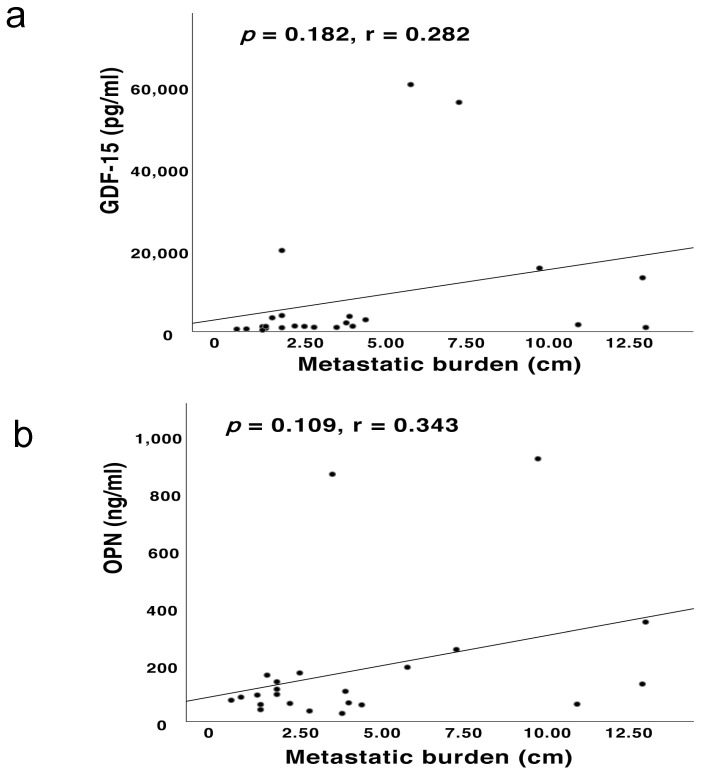
(**a**,**b**) The Pearson’s correlations between the metastatic burden and the blood-based levels of GDF-15 and OPN, respectively.

The present results also revealed moderate correlations between the metastatic burden and the blood-based levels of other two cytokines (i.e., serum GDF-15, *r* = 0.282, *p* = 0.182; plasma OPN, *r* = 0.343, *p* = 0.109) (see Figure 2a,b).

### 3.3. Receiver Operator Characteristic (ROC) Curve Analyses for UM Patients with Metastases Compared with Those without Metastases and Healthy Individuals

**Figure 3:**
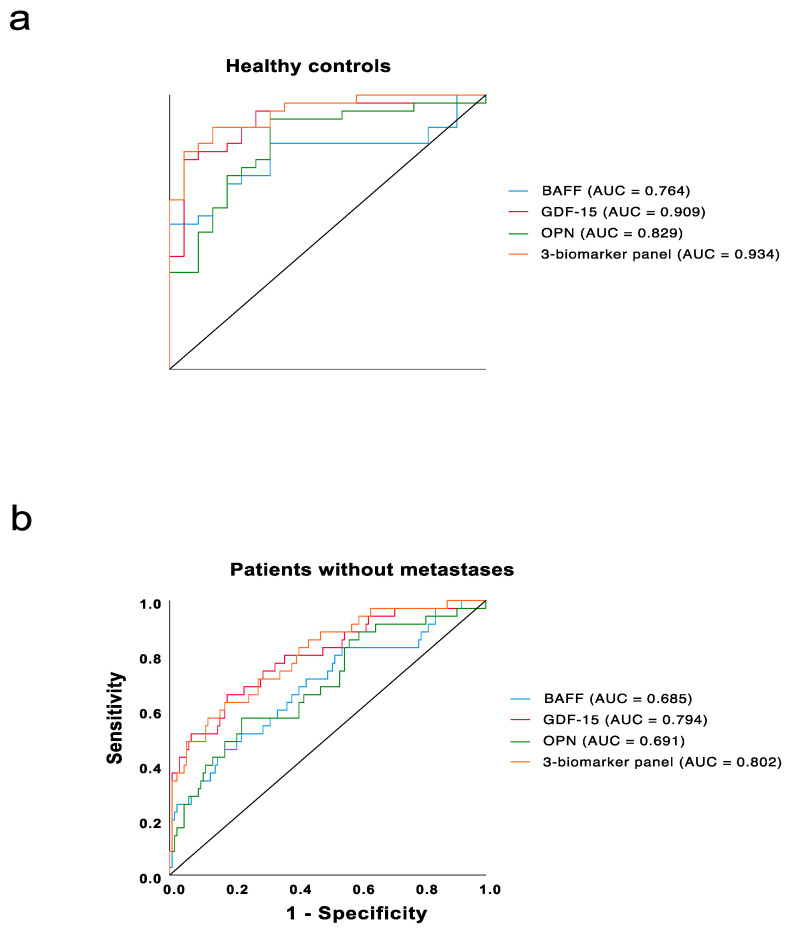
(**a**,**b**) ROC curves for single marker and the combination of three markers in the metastatic UM patients compared with healthy individuals and UM patients without metastases, respectively.

We integrated the data of BAFF [9] with GDF-15 and OPN, and conducted ROC analyses for the levels of the three biomarkers of metastatic patients compared to healthy individuals. As shown in Figure 3a, the three-marker panel outperformed any single biomarker with an impressive AUC of 0.934, followed by GDF-15 with an AUC of 0.909, OPN with an AUC of 0.829 and BAFF with an AUC of 0.764.

Furthermore, ROC analysis was also performed to compare the metastatic patients with those patients without metastases. As shown in Figure 3b, GDF-15 had the best performance with an AUC of 0.794, followed by OPN with an AUC of 0.691 and BAFF with an AUC of 0.685. Notably, the combination of these three markers offered a better performance than a single marker, with an AUC of 0.802. By maximizing the Youden’s index, the plasma OPN level of 92 ng/mL (*J* = 0.363, specificity = 76.3%, sensitivity = 60.0%) and the serum GDF-15 level of 1209 pg/mL (*J* = 0.508, specificity = 82.2%, sensitivity = 68.6%) were identified to have the best performance for distinguishing the metastatic patients from non-metastatic patients [22]. Besides, our previous work on BAFF already indicated the serum BAFF concentration of 1120 pg/mL (*J* = 0.304, specificity = 83.2%, sensitivity = 47.2%) to be the cutoff value [9].

### 3.4. The Correlations among the Blood-Based Levels of BAFF, GDF-15 and OPN

**Figure 4:**
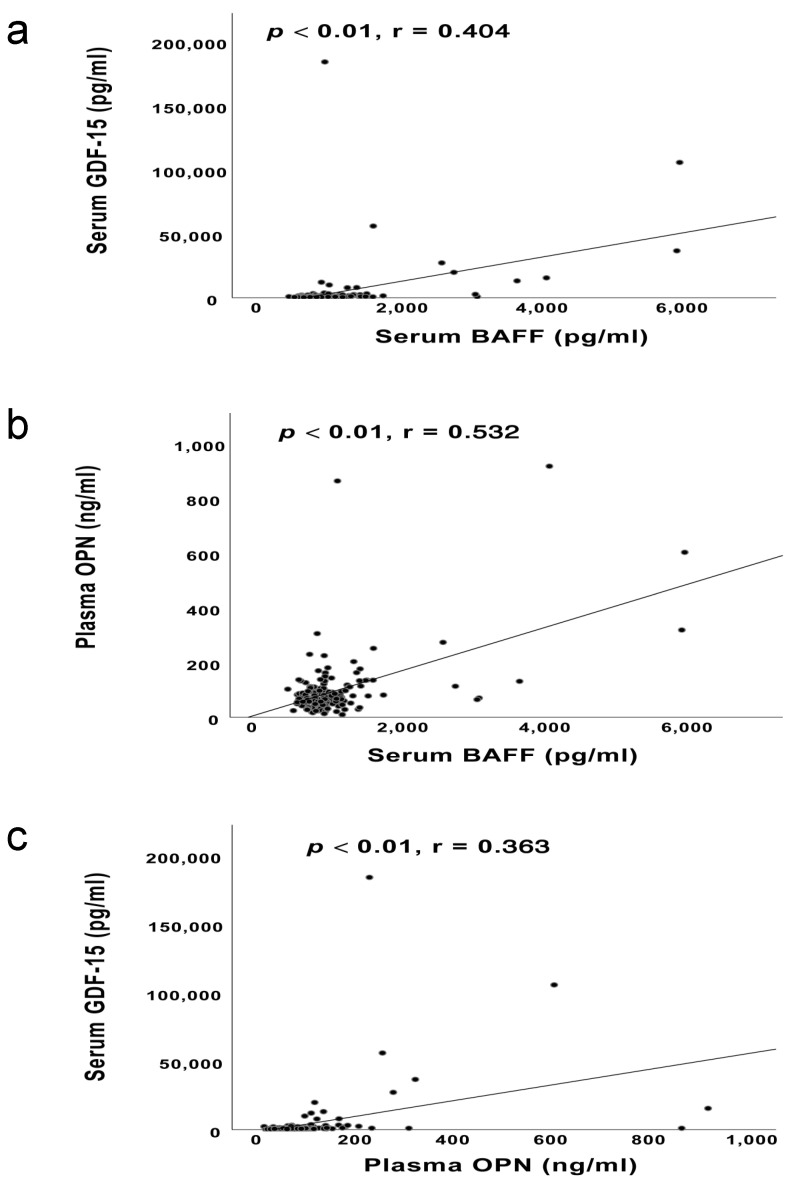
(**a**) The Pearson’s correlations between serum BAFF levels and serum GDF-15 levels; (**b**) The Pearson’s correlations between serum BAFF levels and plasma OPN levels; (**c**) The Pearson’s correlations between plasma OPN levels and serum GDF-15 levels.

Pearson’s correlation coefficients were conducted to study these three cytokines. We observed a remarkable correlation between serum BAFF levels and plasma OPN levels (*p* < 0.01, *r* = 0.532, see Figure 4b). Besides, the statistical analyses on the other two pairs, i.e., serum BAFF and GDF-15 levels (*p* < 0.01, *r* = 0.404, see Figure 4a), plasma OPN and serum GDF-15 levels (*p* < 0.01, *r* = 0.363, see Figure 4c) also revealed significant positive correlations.

### 3.5. The Application of the Cutoff Values

Furthermore, the cutoff values of three biomarkers were introduced to briefly assess the first specimen after imaging metastatic diagnosis of the 36 metastatic patients (see Table 1). The results showed that 63.9% of the patients were identified to have enhanced level of serum GDF-15, while 55.6% and 44.4% of the patients had elevated concentrations of plasma OPN and serum BAFF, respectively. Remarkably, 83.3% of the patients demonstrated increased expression in at least one of these three biomarkers.

**Figure 5:**
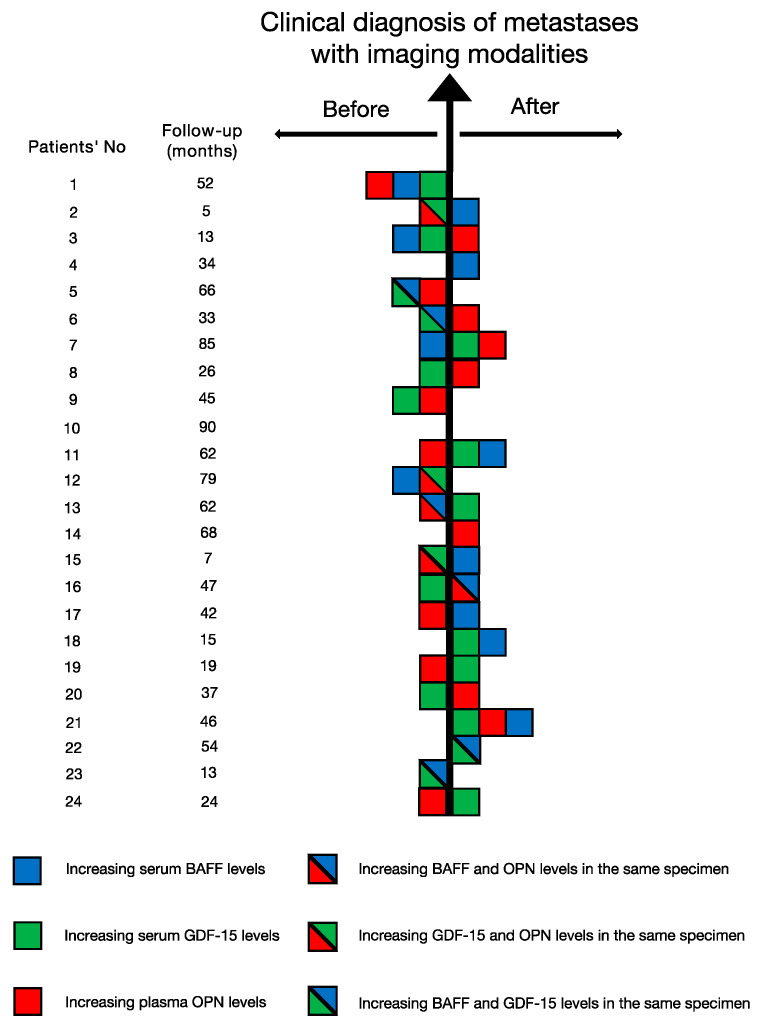
The timeline concerning the upregulated levels of blood-based BAFF, OPN and GDF-15 and the clinical diagnosis of metastases in 24 UM patients.

Since our data bank also contains the blood specimens acquired before the clinical diagnosis of metastases of 24 metastatic patients, we also explored this subgroup to determine whether the upregulated expression of these three biomarkers appeared before the diagnosis of metastasis by conventional imaging modalities. The time interval between the first and last blood acquisition was regarded as the follow-up period. The mean follow-up was identified to be 42.7 months, with a range from 5 to 90 months (see Figure 5). The abovementioned cutoff values of three cytokines were applied to identify the significant increasing expression of these biomarkers. Among 24 patients, only one patient (Patient’s No.: 10) didn’t show any elevation in three cytokines, while the other 23 presented elevated levels in at least one cytokine (see Figure 5). Moreover, 75% of the patients (18 out of 24) demonstrated upregulated expression in at least one of the three biomarkers before the imaging diagnosis of metastases. Besides, by assessing the color of the most left square of each row in Figure 5, we also compared the three biomarkers with the aim to find the cytokine whose upregulated expression appeared at the earliest timepoint. GDF-15 was confirmed to be the earliest appearing elevated biomarker in 11 patients, followed by BAFF with 10 patients and OPN with 8 patients (see Figure 5).

### 3.6. The Kinetic Development of Three Biomarkers

**Figure 6:**
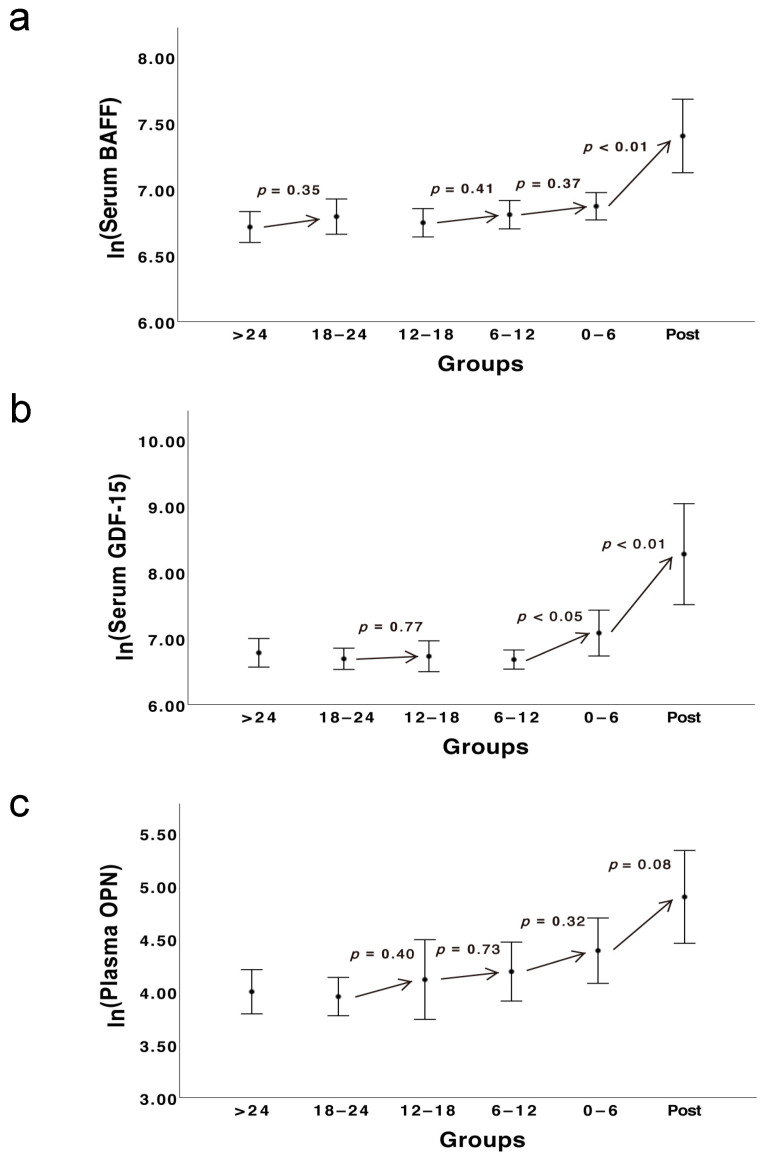
The trends of three biomarkers in a 24-month period before clinical diagnosis of metastatic UM in 24 patients. The blood specimens were divided into six groups according to the acquiring time before the diagnosis of metastases, i.e., >24 months, 18–24 months, 12–18 months, 6–12 months, 0–6 months and those acquired after the metastases (= Post). The arrows show the increasing trends between two adjacent groups. (**a**–**c**) The black dots show the mean of the natural logarithm transformed blood-based levels of BAFF, GDF-15 and OPN, respectively. The error bars represent the 95% confidence interval of the mean.

In Figure 6a, we noticed increasing trends of the serum BAFF levels in four pairs, i.e., >24 and 18–24 months (Student’s *t*-test, *p* = 0.35), 12–18 and 6–12 months (*p* = 0.41), 6–12 and 0–6 months (*p* = 0.37), 0–6 months and the post-metastatic group (*p* < 0.01). For GDF-15, the uprising trends were only observed in three pairs, i.e., 18–24 and 12–18 months (*p* = 0.77), 6–12 and 0–6 months (*p* < 0.05), 0–6 months and the post-metastasis group (*p* < 0.01) (see Figure 6b). With respect to OPN, despite the decreasing trend between the first pair (>24 and 18–24 months), the levels of plasma OPN of the following five groups increased consecutively and the last group had the largest mean value (see Figure 6c). Notably, in all three biomarkers, the pair 0–6 months and the post-metastatic group had the steepest trend slope when compared with the other four pairs.

### 3.7. Bioinformatics Study with TCGA-UVM and GSE84976

**Figure 7:**
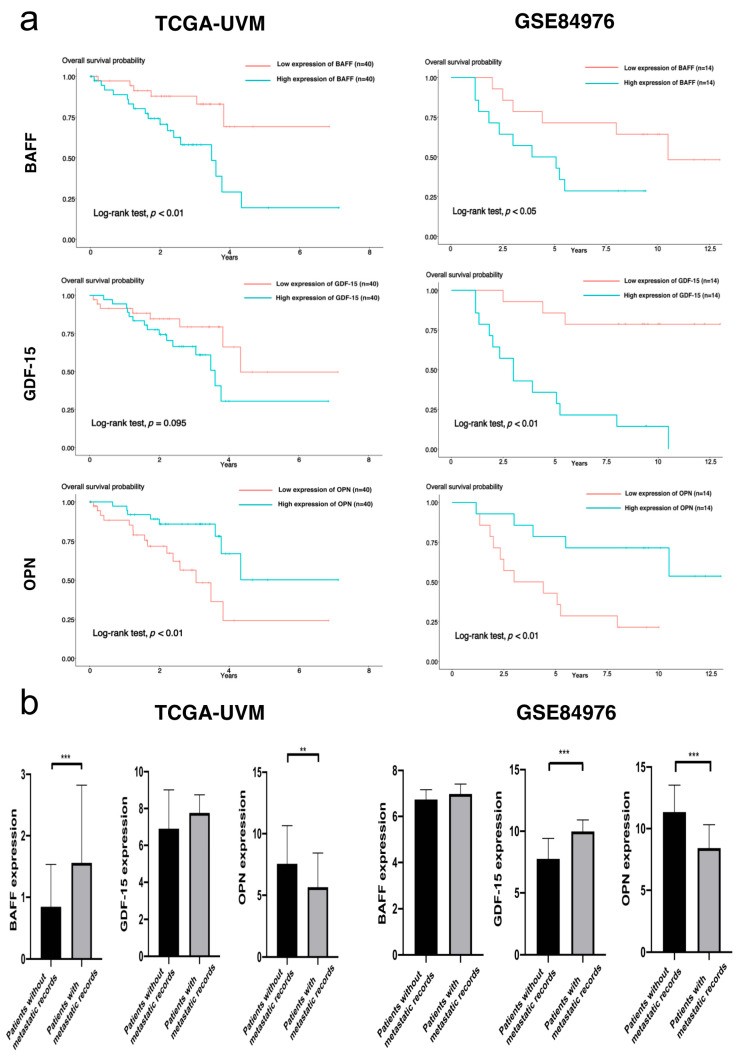
(**a**) Kaplan–Meier survival analysis of high and low gene expression of three markers in two external datasets (TCGA-UVM and GSE84976), log-rank tests were used to check the *p*-values; (**b**) Bar plots showing the gene expression of three markers in TCGA-UVM and GSE84976. In each bar plot, the Student’s *t*-test was conducted to compare the transcriptomic levels of three markers between patients without metastatic records and those with metastatic records. Note: *** means *p* < 0.01, ** means *p* < 0.05.

Additionally, we also explored external cohorts TCGA-UVM (*n* = 80) and GSE84976 (*n* = 28) to study the prognostic value of the gene expression of three markers. As shown in Figure 7a, the high expression of *BAFF* and *GDF-15* in primary UM tissues were identified to be associated with poor overall survival rates in two cohorts. However, the high expression of *OPN* was identified to be a favorable prognostic factor. We also assessed the gene expression of three markers between the patients with and those without metastatic records. Metastatic status was available in 76 patients (26 with and 50 without metastatic records) of TCGA-UVM and in 25 patients (13 with and 12 without metastatic records) of GSE84976. Though metastatic patients had higher levels of *BAFF* and *GDF-15* than non-metastatic patients, they demonstrated lower gene expression of *OPN* (see Figure 7b).

## 4. Discussion

In the current work, we aimed to analyze a biomarker panel consisting of three blood-based proteins to distinguish the UM patients with metastases from those without. First, we have compared the biomarker performance of the three single cytokines BAFF, GDF-15 and OPN in the screening and monitoring of metastases of UM in the same set of patients. The results demonstrated clearly that the expression of these three proteins was significantly higher in patients with metastases than those without metastases. Among the metastatic patients, the levels of these proteins were found to have moderate correlations with the metastatic burden. On the basis of these data we concluded that these three biomarkers were qualified to be integrated into a three-marker panel, to analyze if the combination of these biomarkers would be a better tool for metastases detection.

The usage of this combination model consisting of the three biomarkers was confirmed to outperform a single biomarker in distinguishing the metastatic patients with an AUC of 0.802 in comparison to the single markers with an AUC of 0.794 in case of GDF 15, an AUC of 0.691 in case of OPN and an AUC of 0.685 in case of BAFF.

Only a few studies investigated the levels of biomarkers at consecutive visits, and most studies were performed at two time points (i.e., before and after the clinical confirmation of metastases) [14,16,23,24]. In comparison with the conventional imaging modalities, the majority (75%) of the tested patients demonstrated upregulated expression in at least one of the three biomarkers at an earlier timepoint. These alerting signals may prompt clinicians about the patients with early metastases. Therefore, the regular measurement of these cytokines might be of paramount significance to UM patients. In case of abnormally higher levels, further examination methods such as PET-CT, more small meshed examinations or biopsy might be conducted. As only 24 patients developing metastases in the course were analyzed here, the validation of our findings is still essential by employing larger cohorts. With respect to the elevation dynamics, we observed the increasing trends in the serum levels of three biomarkers in the two-year period before the clinical diagnosis of metastatic diseases. Notably, the levels of three proteins revealed the steepest trend slope between the pair 0–6 months and the post-metastatic period, which might suggest a close association between the appearance of metastatic lesions and the blood-based expression of these biomarkers. Since the current work is a single center-based study, only a limited number of patients developing metastases were studied here; the cutoff values of three biomarkers may not be sufficient and accurate enough to discriminate the patients with metastases. According to Barak and colleagues, the increasing trends of the levels of certain blood-based proteins should be considered too, regardless of whether the expression was above the cutoff values or within the normal range [16,25]. Therefore, despite the elevation of the levels above the cutoff values, the continuous increasing trend of these biomarkers within the normal range should also be considered significant to explore.

According to previous studies, these three biomarkers had distinct biological mechanisms. As its name suggests, BAFF (B-cell activating factor) plays an important role in the immune functions [26]. BAFF was able to promote the activities of B cells, CD4^+^ and CD8^+^ T cells [27,28]. Prior reports indicated that the tumor-infiltrating immune cells played a key role in the metastatic process of UM [18,29,30,31,32]. Besides the immune-associated mechanism, the *BAFF* gene was also reported to participate in the progression of malignancies through interacting with pluripotency-associated genes and epithelial mesenchymal transition (EMT)-associated genes [33,34]. OPN was identified to exist in the malignant cells in two forms, i.e., secretory OPN (sOPN) and intracellular/nuclear OPN (iOPN) [35]. These two forms were found to promote the metastasis with pleiotropic roles. Previous studies on hepatocellular carcinoma, breast and colorectal cancers showed that sOPN could contribute to the metastasis through enhancing EMT-related transcription factors such as TWIST1, SNAI1 and SNAI2 and then decreasing the adhesion of tumor cells [36,37,38]. Moreover, studies on ovarian and breast cancer revealed that OPN could promote the expression of HIF-1α in a PI3k/AKT pathway-dependent manner and then regulate *TWIST1* gene to monitor EMT [39]. By contrast, iOPN was identified to trigger the MET (mesenchymal–epithelial transition) process to induce metastatic colonization. Interestingly, VEGF was proved to regulate the KDR/PLCγ/PKC pathway to facilitate the translocation of sOPN into iOPN [40]. Previous studies on GDF-15 suggested its divergent roles in cancer metastasis. On the one hand, it was identified to promote apoptosis and to suppress angiogenesis and tumor progression [41,42]. On the other hand, its pro-metastatic function was observed in studies on gastric cancer and melanoma [43,44]. Kalli and colleagues studied pancreatic cancer and found that GDF-15 expression was upregulated in the metastatic process through the Akt/CREB1 pathway [45]. Other investigations on prostate and colorectal cancer indicated that GDF-15 promoted metastasis through cooperating with EGR1 or TGFβ-mediated EMT [46,47]. These pathways should be of value to be analyzed in further studies on UM. Compatible with the increase of BAFF and GDF-15 levels with the clinical detection of metastases in our blood-based study, the high expression of gene *BAFF* and *GDF-15* in the primary UM tissues was confirmed to be significantly associated with poor survival of UM patients in cohorts TCGA-UVM and GSE84976. However, the high expression of gene *OPN* was identified to play a different role. The detailed mechanism underlying this inconsistency remains to be elucidated in further studies.

Despite their varied biological bases, we observed moderate correlations among the levels of these three cytokines in the current work (see Figure 4a–c). Thus, these three biomarkers might not be totally independent from each other and are very likely to have some undiscovered associations. Compatible with our hypothesis, several observations have implied the links among them. For instance, GDF-15 was reported by Kim et al. to suppress the expression of OPN [48]. Moreover, an investigation on mice suggested the possible association between GDF-15 and BAFF [49]. Besides, a study on autoimmune diseases indicated that BAFF would promote the secretion of OPN in B cells to maintain the survival of T cells [50]. Otherwise, the three biomarkers may be involved in the metastatic evolution in different ways, and might assume relevant roles in this regard because their levels increase before or with the advent of metastases; however, this increase is not always in parallel. Finding the optimal tumor-biomarker combination has always been a big challenge for oncological studies. According to Borrebaeck, the well-characterized combination should be consisted of independent “orthogonal biomarkers” whose information could optimally contribute to the performance of the combination model [51]. As our study indicated significant correlations between BAFF, GDF-15 and OPN, they might have synergistic contribution to the combined panel. Therefore, further relevant studies are still needed to provide a more perfect panel.

This work has the following limitations. First, the relatively small and unbalanced sample size of different groups may affect the statistical analysis. Second, as the current study is a retrospective research, we are not able to accurately assess the real performance of this three-marker panel in clinical practice. Further prospective studies should be carried out with well-designed strategies to quantify the efficacy of this multi-panel in detecting UM metastases. Third, the conclusions of this work are based on data from a single center; thus, the lack of validation in other cohorts may limit the generalizability of our findings. Fourth, our ophthalmic center focused on treating primary UM patients, while the metastatic UM patients were majorly treated in other specific centers. Due to the lack of data of those metastatic patients, this study is not able to investigate the kinetics of three markers in terms of different treatments against metastatic diseases.

## 5. Conclusions

Taken together, the current study strengthens the evidence base that BAFF, GDF-15 and OPN are promising biomarkers for the early metastases of UM. Besides, a combination panel of BAFF, GDF-15 and OPN will provide a better approach to detect the metastases than utilizing a single biomarker. As metastasis is a complex process, numerous cytokines were supposed to participate in the procedure. Thus, further studies are still needed to explore the early metastases of UM and discover more biomarkers of interest to identify patients who might benefit from early intervention. Since this work is a pilot retrospective study, in-depth prospective researches employing novel technologies are always warranted to offer more insightful and complete elucidations for our findings.

## Figures and Tables

**Table 1:** Application of the cutoff values of three biomarkers in 36 metastatic patients.

Biomarkers	Number of Patients
Serum BAFF ↑	16 out of 36 (44.4%)
Serum GDF-15 ↑	23 out of 36 (63.9%)
Plasma OPN ↑	20 out of 36 (55.6%)
Either serum BAFF ↑, or serum GDF-15 ↑, or plasma OPN ↑	30 out of 36 (83.3%)

Note: ↑ means that the levels of the corresponding biomarkers are higher than their cutoff values.

## Data Availability

The gene sequencing and clinical data of TCGA-UVM and GSE84976 were downloaded on 27 April 2021 with R packages TCGAbiolinks (version 2.12.6) and GEOquery (version 2.54.1).
